# Initial and Sustained Attentional Bias Toward Emotional Faces in Patients with Major Depressive Disorder

**DOI:** 10.3390/jemr18060072

**Published:** 2025-12-01

**Authors:** Hanliang Wei, Tak Kwan Lam, Weijian Liu, Waxun Su, Zheng Wang, Qiandong Wang, Xiao Lin, Peng Li

**Affiliations:** 1 The First Affiliated Hospital of Xinxiang Medical University, Xinxiang 453199, China; weihanliang2006@126.com; 2 National Clinical Research Center for Mental Disorders, Peking University Sixth Hospital, Peking University Institute of Mental Health, Beijing 100191, China; weijian191954@stu.pku.edu.cn; 3 Department of Psychology, The Chinese University of Hong Kong, Hong Kong, China; 1155254047@link.cuhk.edu.hk; 4 Danish Research Centre for Magnetic Resonance, Department of Radiology and Nuclear Medicine, Copenhagen University Hospital Amager and Hvidovre, 2650 Hvidovre, Denmark; 5 Beijing Key Laboratory of Applied Experimental Psychology, National Demonstration Center for Experimental Psychology Education, Faculty of Psychology, Beijing Normal University, Beijing 100875, China; suwaxun@bnu.edu.cn (W.S.); 17372049746@163.com (Z.W.); wangqd@bnu.edu.cn (Q.W.)

**Keywords:** major depressive disorder, attentional bias, eye-tracking, emotional processing, temporal dynamic

## Abstract

Major depressive disorder (MDD) represents a prevalent mental health condition characterized by prominent attentional biases, particularly toward negative stimuli. While extensive research has established the significance of negative attentional bias in depression, critical gaps remain in understanding the temporal dynamics and valence-specificity of these biases. This study employed eye-tracking technology to systematically examine the attentional processing of emotional faces (happy, fearful, sad) in MDD patients (*n* = 61) versus healthy controls (HC, n = 47), assessing both the initial orientation (initial gaze preference) and sustained attention (first dwell time). Key findings revealed the following: (1) while both groups showed an initial vigilance toward threatening faces (fearful/sad), only MDD patients displayed an additional attentional capture by happy faces; (2) a significant emotion main effect (F (2, 216) = 10.19, *p* < 0.001) indicated a stronger initial orientation to fearful versus happy faces, with Bayesian analyses (BF < 0.3) confirming the absence of group differences; and (3) no group disparities emerged in sustained attentional maintenance (all *p*s > 0.05). These results challenge conventional negativity-focused models by demonstrating valence-specific early-stage abnormalities in MDD, suggesting that depressive attentional dysfunction may be most pronounced during initial automatic processing rather than later strategic stages. The findings advance the theoretical understanding of attentional bias in depression while highlighting the need for stage-specific intervention approaches.

## 1. Introduction

Depression is one of the most common mental illnesses, which affects around 5% of adults globally [1]. Understanding its pathophysiology is essential for advancing effective treatments. Cognitive models highlight attentional biases toward emotional stimuli as a core mechanism in depression [2]. These biases—defined as the selective prioritization of specific stimuli while disregarding others—serve as a well-established marker of functional abnormalities in major depressive disorder [3]. These biases—the selective prioritization of certain classes of information—are well-established markers of dysfunction in MDD and represent a promising target for therapeutic intervention [4,5]. Recent meta-analytic evidence suggests that modifying negative attentional biases can reduce the depressive symptom severity, albeit with modest effect sizes [6].

The dot-probe task is a widely used paradigm for assessing attentional biases [7]. In this task, participants are presented with pairs of stimuli—one neutral and one emotional—followed by a probe (e.g., a dot) appearing in the location of one stimulus [8]. Response times (RTs) to the probe indicate attention allocation, with faster responses suggesting preferential attention to the preceding stimulus. However, RT-based measures have limitations in capturing the dynamic nature of attention [9], as biases may emerge at different processing stages (e.g., initial orienting vs. sustained attention) [10]. In contrast, eye-tracking offers a more reliable and continuous method for assessing visual attention, allowing a detailed analysis of attentional processes over time and enhancing our understanding of attentional dynamics [11,12]. The eye-tracking method—which can reveal several eye movement components, including early orienting, maintenance, and biases away from stimuli—is considered more reliable than response time-based tasks, such as the dot-probe paradigm [13,14]. By recording gaze patterns, researchers can distinguish between early attentional orienting, sustained attention, and avoidance behaviors—components that RT measures alone cannot fully capture [2]. Given these advantages, eye-tracking has become an increasingly valuable tool in depression research. The attentional profile of MDD is characterized not by a hyper-vigilance that initially seeks out threats [2] but by a pattern of maintenance that gets “stuck” on negative information and “avoids” positive information once it has been encountered [15]. Existing studies using eye-tracking have consistently demonstrated that individuals with MDD exhibit an attentional bias toward negative stimuli [16,17]. Regarding positive emotions, studies have found that MDD patients also showed a significant and moderate bias away from positive stimuli [5,6]. However, whether the diminished attention occurs in the early or late phase remains unclear. Kellough et al. [18] found that individuals with MDD showed diminished attention toward happy faces during the sustained phase, with a 30 s period without an initial bias. However, Ao et al. [5] found that individuals with MDD showed a diminished attentional bias toward happy faces during the initial phase by the EEG P1 component. Regarding eye-tracking, the meta-analysis also found no consistent evidence that patients with MDD have any abnormality during the initial orientation phase [19]. These mixed findings highlight the need for a more systematic investigation into the nature and time course of positive stimulus processing in depression.

While a substantial body of studies has documented sustained attentional biases toward negative stimuli in MDD [20,21], the literature on the processing of positive stimuli is markedly less consistent, creating a significant gap in the cognitive model of depression. Some eye-tracking studies suggest that MDD may be associated with diminished attentional engagement with positive stimuli, such as happy faces [22,23]. Conversely, other findings indicate that this blunted positive bias may not be evident in subclinical groups or may even reverse as a form of strategic, effortful emotion regulation [24,25,26]. This inconsistency points to a potentially critical moderating factor: severity.

The temporal locus of this diminished positive bias is also unclear. Neurophysiological studies using EEG suggest early deficits in the initial orienting toward happy faces [5], while other eye-tracking studies find abnormalities only in later, sustained attention phases without early orienting differences [27]. A meta-analysis further underscores this confusion, finding no consistent evidence for abnormalities in the initial orienting phase in MDD patients when using eye-tracking [4]. This unresolved contradiction highlights the need for a study designed to explicitly separate and measure these distinct attentional phases within the same experimental framework.

Traditional RT-based dot-probe tasks can conflate different cognitive stages, and free-viewing tasks allow for strategic processing that may obscure early attentional dynamics. To address these limitations, our study adopted a gaze-contingent, variable-start paradigm, in which the fixation cross was presented randomly to the left, middle, or right, and a fixation was required to trigger the stimulus onset. This design was explicitly intended to isolate the earliest, automatic phase of attentional orienting by controlling the starting position of the gaze, thereby reducing anticipatory or strategic influences. Our confirmatory analyses tested the effects of the group (MDD, HC) and emotion (happy, fearful, sad) on two primary outcomes: initial orienting (initial gaze preference) and sustained attention (first dwell time). We specifically tested for a group × emotion interaction on both outcomes. Exploratory analyses followed up on these effects to characterize the nature of attentional patterns, particularly for happy faces, in each group. Specifically, we aim to compare attentional patterns toward emotional faces (happy, sad, and fearful) between MDD patients and healthy controls, dissect the time course of these biases by distinguishing early orienting (initial gaze preference) from sustained attention (first dwell time), and investigate the relationship between biases toward negative and positive stimuli to clarify whether a reduced positive bias is an independent feature of MDD or is intrinsically linked to a negative attentional bias. Based on the existing literature and these aims, we hypothesize a significant main effect of emotion on the initial orienting (initial gaze preference), with all participants showing a general vigilance toward threat-related (fearful) faces. We predict a significant group (MDD vs. HC) × emotion interaction with the initial orienting. wherein individuals with MDD will show a reduced first-gaze preference for happy faces and a heightened first-gaze preference for sad faces compared to healthy controls. Exploratorily, we anticipate a significant group × emotion interaction with the initial gaze latency (TTFF). Compared to healthy controls, individuals with MDD will show faster latencies (greater attentional capture) for sad faces and slower latencies (reduced capture) for happy faces. For sustained attention, we expect a significant group × emotion interaction, as measured by the first dwell time and total dwell time. individuals with MDD will show shorter dwell times on happy faces and longer dwell times on sad faces. Finally, we hypothesize that within the MDD group, attentional bias scores for sad and happy faces will be significantly negatively correlated. This will provide evidence that a stronger bias toward sadness is associated with a stronger bias away from happiness, suggesting these are linked features of the disorder. By testing these hypotheses, we will elucidate the precise nature and time-course of attentional abnormalities in MDD, which may contribute to the development of more targeted interventions.

## 2. Methods

### 2.1. Participants

A total of 127 participants were initially recruited. The final sample consisted of 61 adults with major depressive disorder (MDD) (Mage~ = 25.97 years, SD = 6.43; 15 males) and 47 healthy controls (HC) (Mage~ = 26.09 years, SD = 4.13; 15 males) (See Table 1). The groups were matched for sex, ethnicity, marital status, and education level. The majority of participants were Han Chinese, and the number of ethnic minority participants was balanced between the MDD and HC groups.

MDD Group Recruitment and Criteria: Participants with MDD were recruited from hospital outpatient services. Inclusion required the following: (1) a DSM-IV diagnosis of MDD; (2) a score of ≥17 on the 17-item Hamilton Depression Rating Scale (HAMD-17); (3) no comorbid DSM-IV Axis I disorders (e.g., schizophrenia, bipolar disorder, substance dependence); and (4) no history of significant chronic physical or neurological illness. The mean score of HAMD in the MDD group is 24.30. Mean of Hamilton Anxiety Rating Scale (HAMA) is 24.24.

HC Group Recruitment and Criteria: Healthy controls were recruited online and screened to ensure (1) no current or past DSM-IV Axis I disorders; (2) a HAMD-17 score < 7; and (3) no history of significant chronic physical or neurological illness. The mean score of HAMD in the HC group is 4.46. The mean of HAMA is 5.49.

### 2.2. Participant Exclusion

Of the initially recruited 76 MDD participants, 15 were excluded: 10 were diagnosed with bipolar disorder upon further clinical interview, 2 did not meet the HAMD-17 score threshold, 1 was excluded due to poor eye-tracking data quality (see Data Preprocessing), and 2 had no valid trials in at least one experimental condition. Of the 51 initially recruited HC participants, 4 were excluded prior to analysis due to emerging mental health concerns. This resulted in the final sample of 61 MDD and 47 HC participants.

### 2.3. Power Justification

An a priori power analysis was conducted using GPower 3.1 [28] to determine the minimum sample size required to test our primary hypothesis of a group × emotion interaction with initial orienting (initial gaze preference). The analysis was based on an estimated effect size of f = 0.35 (a conventional medium effect size for interactions in psychological research), an α error probability of 0.05, and a desired power (1-β) of 0.8 for a repeated measures ANOVA within–between interaction. The analysis indicated a required total sample size of 93 (See Supplementary Figure S2 for details). Our final sample of 108 participants (61 MDD, 47 HC) exceeds this requirement, providing adequate power (>0.8) to detect the hypothesized effect.

### 2.4. Materials

**Figure 1:**
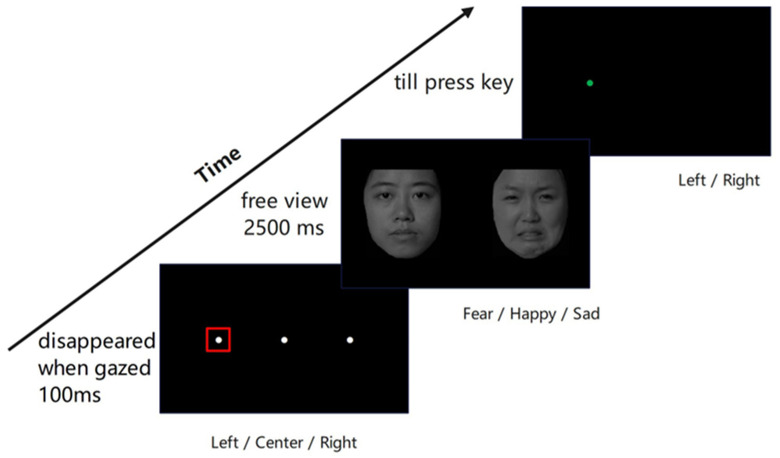
Experimental procedure for dot-probe task of eye-tracking. At the beginning of the task, a white dot was on the screen, which disappeared when the participants’ gaze lasted 100 ms within a square region of 2° × 2° (i.e., the red squared region). Then, a pair of emotional–neutral faces appeared for 2500 ms, after which the target appeared. Participants had to identify whether the green dot appeared on the left or right side of the screen by pressing the corresponding keys.

The stimulus included 24 Chinese faces with different expressions, including four happy, four sad, four fearful, and twelve neutral expressions with balanced male and female faces from the Chinese Facial Affective Picture System (CFAPS) [29,30]. All images were removed of hair (Figure 1), rendered in grayscale, and controlled for luminance using the SHINE toolbox in Matlab [31]. One emotional face was randomly paired with one neutral face, with each pair of faces matched for gender. We used three types of face pairs, fear–neutral, happy–neutral, and sad–neutral, with 4 pairs for each type, for a total of 12 pairs.

### 2.5. Procedure

The task was presented on a 37.5 cm × 30 cm 19-inch LCD monitor with a 1024 × 768 pixel resolution. We used Python (version 3.8.12) and pylink [32] for eye-tracking data recording and stimulus presentation. Participants sat about 66 cm away from the monitor, with an Eyelink eye tracker at 500 Hz recording their eye movements (Eyelink 1000, SR Research, Ottawa, ON, Canada). After six trials of practice, participants completed a five-point calibration procedure. The procedure would repeat until the eye achieved good mapping on all five test positions (smaller than 1.5 degrees of maximum visual angle error and below 1.0 degrees for the averaged visual angle error).

The experiments used an adapted version of the eye-tracking dot-probe task (Figure 1) from [33]. In their experiment, a white fixation always started at the center of the screen for 1000 ms, regardless of whether the participant gazed or not. However, in this study, each trial started with a white fixation dot at the left (aligned with the center of the left image), right (aligned with the center of the right image), or center of the screen in a fully randomized order, with each of the three positions appearing equally often (48 trials each) across the experiment. When the participants gazed at the white dot within a visual angle of 2° × 2° for at least 100 ms, a face pair appeared with 18 visual angles apart (from their centers) in size of a 9° × 10° visual angle for 2500 ms, and participants viewed freely. The left/right order of emotional and neutral faces within each pair was presented randomly, with an equal number of trials (72 trials) for each emotional face position (left/right). Then, a target probe (a green dot with a diameter of 1° visual angle) appeared on the left or right side of the image center randomly until a response. Participants needed to press “Z” if the dot was on the left and “/” if on the right. The task consisted of 144 trials, designed as 3 (facial expression: fearful, happy, sad) × 2 (emotional face location: left, right) × 3 (fixation dot position: left, center, right) × 4 (face pair identity) × 2 (repetitions). The order of all trials was fully randomized for each participant. The entire task took approximately 20 min.

### 2.6. Data Analysis

#### 2.6.1. Data Preprocessing

Eye-tracking data were preprocessed using a custom Python pipeline. Data from the Eyelink recorder were exported and processed for each trial. The Eyelink 1000 system used in this study has a typical spatial accuracy of approximately 0.25–0.5° of visual angle under optimal calibration conditions, which is consistent with manufacturer specifications and widely accepted in the field for gaze-contingent paradigms. The median visual angle error in the calibration phase is 0.36 degrees, with 0.18 degrees as the standard deviation (SD) for the HC group. For the MDD group, the median visual angle error is 0.41 degrees, with 0.16 degrees as SD. The max visual angle error is 1.47 degrees. The mean valid trial rate is 94.2% with 5.3% as SD in the HC group and 94.6% with 4.7% as SD for the MDD group. The mean blink/sample loss rate is 6.0% with SD of 7.5% in the HC group and 6.9% with SD of 6.4% in the MDD group. See Table 2 for details. Reaction times to the probe were recorded and analyzed exploratorily (see Supplementary Table S1). However, the extended free-viewing period prior to the probe presentation means RTs capture a later composite of cognitive processes; thus, the eye movement metrics were considered the primary measures of initial orienting and sustained attention for this study.

#### 2.6.2. Eye Movement Data Preprocessing

- Trial Validity and Exclusion: Although a stricter tolerance could be applied, we selected a 4° criterion. This threshold is conservative relative to the system’s spatial accuracy, while minimizing the exclusion of valid trials due to minor oculomotor drift. A trial was considered valid only if the participant’s initial gaze episode during guiding phase was within a 4° × 4° area centered on the initial guidance dot. This ensured the participant’s gaze was correctly aligned at the trial’s start. Participants were required to have valid data for at least 70% of trials in each of the 3 (emotion) × 3 (fixation position) = 9 experimental conditions to be included in the analysis. This stringent threshold ensured that even the condition with the fewest valid trials had sufficient data for reliable analysis. All included participants met this criterion, with the minimum valid trial rate across any condition being 73.2%.
- Analytical Strategy: We employed a hierarchical analytical approach to test our hypotheses. Our primary analyses consisted of mixed-effects models, which are robust to the limitations of traditional ANOVAs for repeated measures data and allow for the inclusion of trial-level data. Secondly, exploratory analyses included one-sample tests, which were corrected for multiple comparisons.
- Eye Movement Indices:
  ○ Initial Gaze Preference (Initial Orienting): For trials where the initial guidance dot was at the screen center, we calculated the proportion of initial gazes directed toward the emotional face versus the neutral face. For our primary analysis of initial orienting, we fitted a generalized linear mixed model (GLMM) with a binomial distribution (logistic regression) to the trial-level binary outcome of the initial gaze (emotional face = 1, neutral face = 0). The model included group (MDD, HC) and emotion (happy, fearful, sad) and their interaction as fixed effects. Random intercepts for subject (subject) and stimulus item (face) were included to account for repeated measures across participants and stimuli. The model was specified as follows: Initial Gaze ~ Group ×Emotion + (1 | subject) + (1 | face). Model fits were assessed by examining residuals.
  ○ As a secondary, exploratory analysis to characterize attentional patterns within each group, we performed one-sample *t*-tests against a test value of 0.5 (chance) on the mean first-gaze preference score for each emotion separately for the HC and MDD groups. To control the false discovery rate (FDR) across these six tests, we applied the Benjamini–Hochberg procedure. We report the adjusted *p*-values and 95% confidence intervals for these exploratory tests.
  ○ Initial Gaze Latency (Time-to-First-Fixation—TTFF): For each valid trial, we calculated the time (in milliseconds) from stimulus onset until the first fixation within the Area of Interest (AOI) of any face (emotional or neutral). To derive a bias score, we subtracted the latency to fixate on the emotional face from the latency to fixate on the neutral face for each trial (TTFF_Emotional—TTFF_Neutral). Negative scores indicate a faster orienting (i.e., attentional capture) toward the emotional face relative to the neutral face. We conducted a repeated measures ANOVA on these difference scores to examine the effects of group and emotion.
  ○ First Dwell Time (Sustained Attention): For all valid trials, we calculated the total duration of the initial gaze episode on each face (emotional or neutral) after the pair was presented, serving as a measure of attentional maintenance. For our primary analysis of sustained attention, we fitted linear mixed models (LMMs) to the log-transformed first dwell time on emotional faces. Visual inspection of Q-Q plots (See Supplementary Figure S1 for details) and a Shapiro–Wilk test on the residuals of a preliminary model confirmed that log-transformation successfully addressed the positive skew in the raw dwell time data (W = 0.98, *p* < 0.001 for raw residuals; W = 1.00, *p* = 0.12 for log-transformed residuals). Levene’s test indicated homogeneity of variance across groups (*p* > 0.05). The model included the same fixed effects (group × emotion) and random intercepts for subject (subject) and trial (trial num) to account for the nested design: log (First Dwell Time) ~ Group × Emotion + (1 | subject) + (1 | Trial num). We report the model estimates, 95% confidence intervals (CIs), and *p*-values based on the Satterthwaite approximation for degrees of freedom.
  ○ Total Dwell Time Difference: To assess overall attentional maintenance and avoidance throughout the entire trial, we also calculated the total dwell time on each face (emotional and neutral) for the full 2500 ms stimulus presentation. A difference score was created for each trial by subtracting the total dwell time on the neutral face from the total dwell time on the emotional face (Total_Dwell_Emotional—Total_Dwell_Neutral). Positive scores indicate sustained attention toward the emotional face, while negative scores indicate avoidance. We conducted a repeated measures ANOVA on these difference scores to examine the effects of group and emotion.

#### 2.6.3. Potential for Deep Learning Enhancements

While the current study relied on conventional gaze metrics and manual calibration, recent advances in deep learning offer promising avenues for enhancing the precision and automation of eye-tracking analyses. For instance, transformer-based architectures have shown strong performance in automated image acquisition and feature extraction tasks, such as in urban imagery analysis [34]. Similar approaches could be adapted to improve fixation detection, classify micro-saccades, or even identify subtle attentional patterns across large datasets, thereby reducing manual preprocessing and increasing reproducibility. Future studies may benefit from integrating such computational methods to further validate and extend eye-tracking findings in clinical populations.

### 2.7. Bayesian Inference

To quantify the evidence for the null hypothesis (i.e., the absence of a group × emotion interaction or a main effect of group), we complemented the frequentist analyses with Bayesian equivalents of the primary models. The Bayesian inference was calculated by the BayesFactor package (version 0.9) in R software (version 4.3.3). To test our primary hypothesis that HC and MDD groups differ significantly, we calculated the difference in posterior distributions for the group effect with 1000 iterations. The Bayes factor (BF) was then calculated to quantify the evidence for or against the hypothesis that HC and MDD groups are different. The BF was computed as the ratio of the probability that the difference is greater than zero to the probability that the difference is less than zero. A BF < 1/3 provides substantial evidence for the null hypothesis (no group difference).

## 3. Results

### 3.1. Initial Attentional Bias: Initial Gaze Preference

The primary generalized linear mixed model (GLMM) revealed a significant main effect of emotion on the likelihood of the initial gaze towards the emotional face (χ^2^(2) = 12.81, *p* = 0.002). However, there was no significant main effect of the group (χ^2^(1) = 0.54, *p* = 0.462) and no significant group × emotion interaction (χ^2^(2) = 2.68, *p* = 0.262). Parameter estimates from the model are presented in Table 3. The intercept represents the log-odds of the initial gaze to an emotional face for the HC group viewing a fearful face. The model indicated that, relative to fearful faces, both groups were less likely to first fixate on happy faces (b = −0.263, SE = 0.073, z = −3.588, *p* < 0.001) and sad faces (b = −0.153, SE = 0.074, z = −2.072, *p* = 0.038). The variance for the random intercepts was near zero, indicating that most of the variance was explained by the fixed effects.

**Figure 2:**
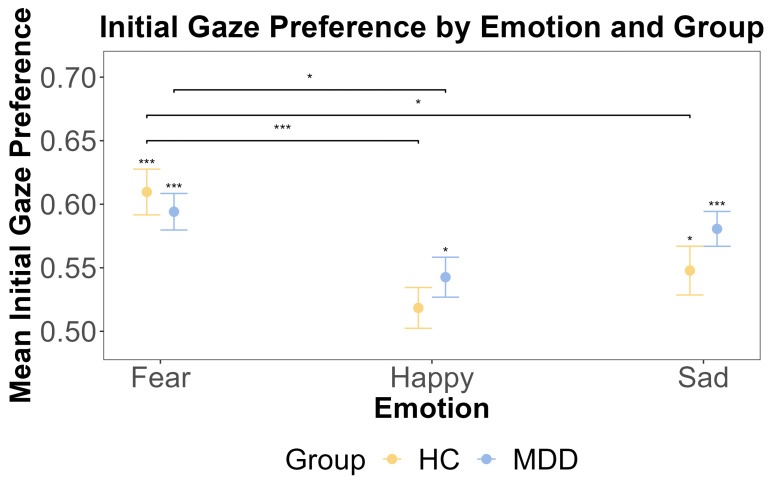
The results of one-sample *t*-tests and the post hoc test for the initial gaze preference by emotion and group. The *x*-axis includes three different emotions: fear, happy, and sad. The *y*-axis is the mean initial gaze preference. The yellow color indicates the HC group, and blue color indicates the MDD group. The error bars represent the standard error of the mean. Statistical significance (one-sample *t*-test against a test value of 0.5) is indicated by asterisks: * *p* < 0.05, and *** *p* < 0.001.

Supported by the non-significant interaction in the primary model, we proceeded with exploratory one-sample *t*-tests separately for the HC group and MDD group to examine the initial gaze preference for emotional faces against 0.5. Results showed that the HC group exhibited the initial attentional bias for fearful and sad faces rather than neutral faces (fear: t (46) = 6.09, *p* < 0.001, Cohen’s d = 0.89, Cohen’s d CIs [0.579, Inf]; happy: t (46) = 1.15, *p* = 0.771; sad: t (46) = 2.49, *p* = 0.0495, Cohen’s d = 0.363, Cohen’s d CIs [0.51, Inf]). And the MDD group showed an initial attentional bias for fearful, happy, and sad faces (fear: t (62) = 6.54, *p* < 0.001, Cohen’s d = 0.824 Cohen’s d CIs [0.570, Inf], happy: t (62) = 2.71, *p* = 0.0261, Cohen’s d = 0.341 Cohen’s d CIs [0.516, Inf], sad: t (62) = 5.88, *p* < 0.001, Cohen’s d = 0.741, Cohen’s d CIs [0.558, Inf]). We further used a repeated measures ANOVA to test if there were any main effects of the group (MDD and HC) and emotion (happy, fear, and sad), as well as the interaction between these factors, on the initial gaze preference for the emotional face. The ANOVA showed that the main effect of the group was insignificant (F (1, 108) = 1.006, *p* = 0.318, η^2^G = 0.003). This was further supported by the Bayesian inference for testing the HC and MDD group difference (BF = 0.244). However, the main effect of the emotion was significant (F (2, 216) = 10.189, *p* < 0.001, η^2^G = 0.056), and post hoc tests revealed that the initial gaze preference for fearful faces was significantly higher than for happy faces (t (108) = 4.736, *p* < 0.001). The interaction effect between the group and emotion was insignificant (F (2, 216) = 1.33, *p* = 0.27, η^2^G = 0.008). These results are shown in Figure 2.

### 3.2. Initial Attentional Bias: Initial Gaze Latency (Time-to-First-Fixation)

**Figure 3:**
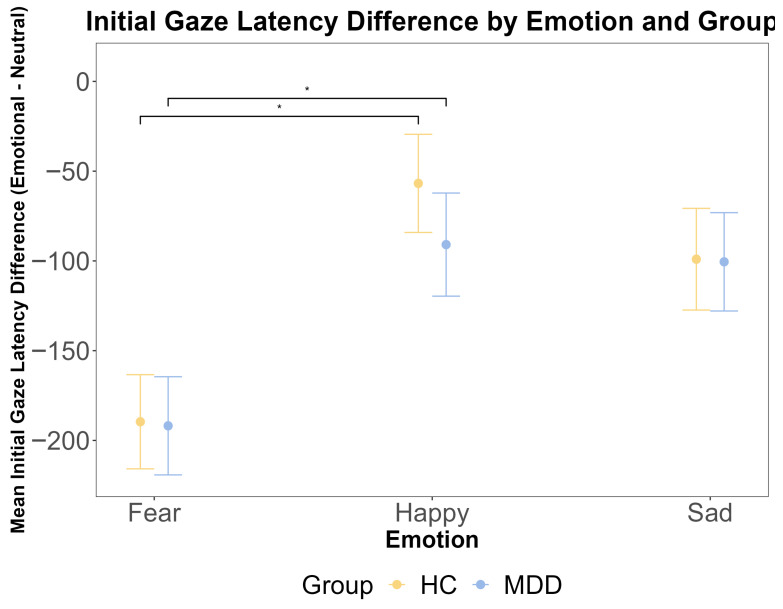
The results of the ANOVA test for the initial gaze latency difference by emotional face latency minus neutral face latency (ms) by emotion and group. The *x*-axis represents three different emotions: fear, happy, and sad. The *y*-axis is the mean initial gaze latency difference (ms). The yellow color indicates the HC group, and the blue color indicates the MDD group. The error bars represent the standard error of the mean. Statistical significance is indicated by asterisks: * *p* < 0.05.

To complement the binary first-gaze preference measure, we analyzed the initial gaze latency (TTFF) as a continuous index of the speed of the attentional orienting. A repeated measures ANOVA on the emotional–neutral latency difference scores revealed a significant main effect of emotion (F (2, 212) = 7.14, *p* = 0.001, η^2^G = 0.043). Post hoc tests confirmed that the attentional capture was significantly stronger for fearful faces compared to happy faces across both groups (*p* < 0.001). However, there was no significant main effect of the group (F (1, 106) = 0.003, *p* = 0.960) and no significant group × emotion interaction (F (2, 212) = 0.20, *p* = 0.819) (Figure 3). Bayesian analyses provided substantial evidence for the null hypothesis of no group difference (BF < 0.3). These results, showing a robust effect of emotional salience on the speed of initial orienting but no modulation by the diagnostic group, converge with the first-gaze preference data to indicate that the earliest automatic capture of attention is intact in MDD.

### 3.3. Attentional Maintenance: First Dwell Time

For attentional maintenance, we calculated the first dwell time on emotional and neutral faces separately. Prior to conducting repeated measures ANOVAs, we confirmed the assumption of sphericity using Mauchly’s Test. For the dwell time on emotional faces, Mauchly’s test was non-significant, with W = 0.938 and *p* = 0.231. For the dwell time on neutral faces, Mauchly’s test was also non-significant, with W = 0.945 and *p* = 0.301. Consequently, sphericity was assumed, and no corrections were applied to the ANOVA results. We conducted separate repeated measures ANOVAs for the first dwell time on emotional and neutral faces to examine whether there were any main effects of the group and emotion and any interactions between these factors. For the emotional face, the ANOVA showed that the main effect of the group was insignificant (F (1, 108) = 0.041, *p* = 0.840, η^2^G < 0.001). This was further supported by the Bayesian inference for testing the HC and MDD group difference (BF = 0.164). The main effect of emotion was also insignificant (F (2, 216) = 1.934, *p* = 0.147, η^2^G = 0.002). The interaction effect between the group and emotion was insignificant (F (2, 216) = 0.36, *p* = 0.69, η^2^G < 0.001).

Given the right-skewed distribution of the dwell time data, we complemented the ANOVA with a linear mixed-effects model on log-transformed dwell times. This model included random intercepts for the subject and trial to account for the nested design. For the dwell time on emotional faces, the model revealed a significant main effect of emotion, driven by significantly shorter dwell times on sad faces compared to fearful faces (b = −0.051, SE = 0.025, t (4801) = −2.086, *p* = 0.037). No other main effects or interactions were significant (all *p* > 0.05). The model for the dwell time on neutral faces showed no significant main effects or interactions (all *p* > 0.05).

### 3.4. Attentional Maintenance: Total Dwell Time Difference

**Figure 4:**
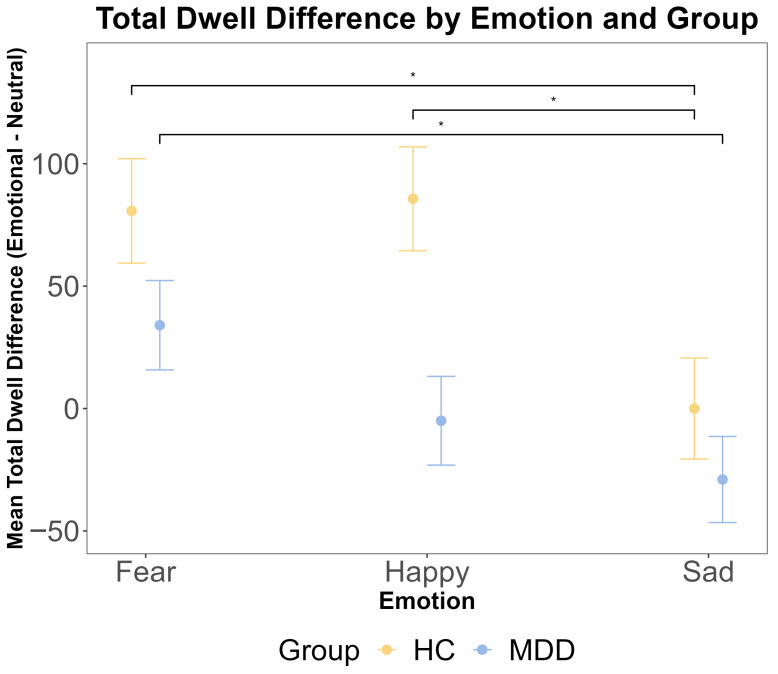
The results of ANOVA test for the total dwell time difference (emotional–neutral face) by emotion and group. The total dwell time was calculated for the entire 2500 ms stimulus presentation period. The *x*-axis includes three different emotional faces that are paired with a neutral face: fear, happy, and sad. The *y*-axis is the mean total dwell time difference (ms). The yellow color indicates the HC group, and the blue color indicates the MDD group. The error bars represent the standard error of the mean. Statistical significance is indicated by asterisks: * *p* < 0.05.

To complement the analysis of the first dwell period, we analyzed the total dwell time difference across the entire trial. A repeated measures ANOVA revealed a significant main effect of emotion (F (2216) = 5.53, *p* = 0.005, η^2^G = 0.023). However, there was no significant main effect of the group (F (1108) = 1.98, *p* = 0.162, η^2^G = 0.005) and no significant group × emotion interaction (F (2216) = 0.97, *p* = 0.380, η^2^G = 0.003). Bayesian analyses provided substantial evidence for the null hypothesis of no group difference (BF < 0.3). Post hoc pairwise comparisons within each group indicated that for HC participants, the total dwell time was significantly longer for fearful faces compared to both happy (t (108) = 2.53, *p* = 0.034) and sad (t (108) = 2.72, *p* = 0.020) faces. For MDD participants, the total dwell time was significantly longer for fearful faces compared to sad faces (t (108) = 2.44, *p* = 0.043) but not compared to happy faces (*p* = 0.475). These results are presented in Figure 4.

**Figure 5:**
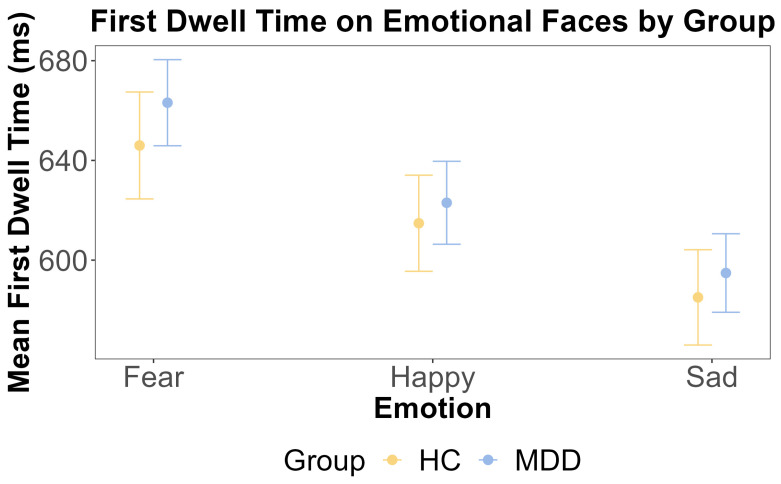
The results of ANOVA test for the first dwell time on emotional faces by emotion and group. The *x*-axis includes three different emotions: fear, happy, and sad. The *y*-axis is the mean first dwell time on the emotional face (ms). The yellow color indicates the HC group, and the blue color indicates the MDD group. The error bars represent the standard error of the mean.

**Figure 6:**
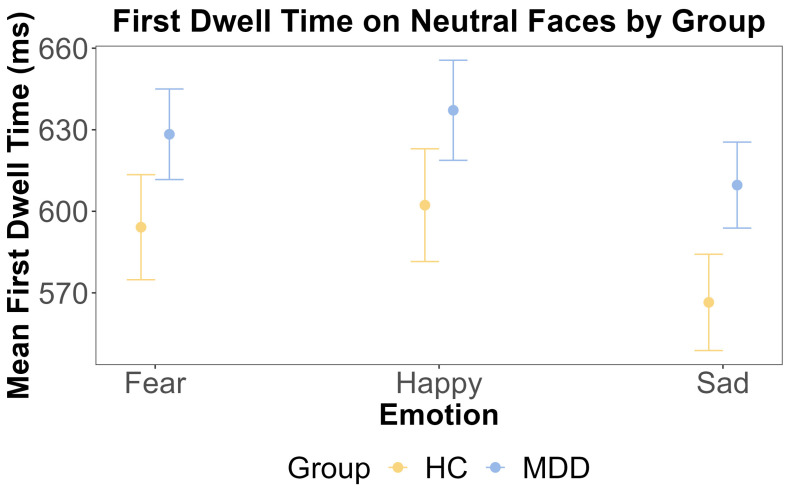
The results of ANOVA test for the first dwell time on neutral faces by emotion and group. The *x*-axis includes three different emotional faces that are paired with a neutral face: fear, happy, and sad. The *y*-axis is the mean first dwell time on the neutral face (ms). The yellow color indicates the HC group, and the blue color indicates the MDD group. The error bars represent the standard error of the mean.

For the neutral face, the ANOVA showed that the main effect of the group was insignificant (F (1, 108) = 0.245, *p* = 0.622, η^2^G = 0.002). This was further supported by the Bayesian inference for testing the HC and MDD group difference (BF = 0.098). The main effect of emotion was also insignificant (F (2, 216) = 1.986, *p* = 0.140, η^2^G < 0.001). The interaction effect between the group and emotion was also insignificant (F (2, 216) = 0.47, *p* = 0.63, η^2^G < 0.001). These results are shown in Figure 5 and Figure 6.

## 4. Discussion

The current study employed eye-tracking to investigate attentional biases in MDD, focusing on both initial orienting and sustained attention stages. Contrary to our specific hypotheses, we found no significant group × emotion interactions, with Bayesian analyses providing substantial evidence for the null hypothesis of no group differences in either the first-gaze preference or dwell time. However, both groups exhibited similar patterns of early attentional vigilance toward threat-relevant stimuli (fearful and sad faces), aligning with prior reports of generalized negativity biases in emotional processing [23,24]. The key novel finding was that MDD participants, unlike HC participants, also demonstrated an initial orienting bias toward happy faces. This finding contrasts with some studies reporting reduced attention to positive stimuli in MDD [5,6] and may be explained by differences in the task design.

The mixed results in the literature regarding positive attentional biases in depression may be moderated by the experimental paradigm. For instance, free-viewing tasks often reveal a sustained avoidance of positive stimuli or a continued focus on negative ones [22,23,27], whereas reaction-time-based dot-probe tasks may conflate multiple cognitive stages. In contrast, our gaze-contingent, centrally initiated dot-probe task specifically isolates the earliest, automatic phase of attentional orienting. By controlling the gaze position and requiring a fixation to trigger stimulus onset, we likely captured a purer measure of the initial attentional capture, less influenced by top-down control. Our findings suggest that individuals with MDD may exhibit generalized hypervigilance to all emotionally salient cues, including happy faces, at the earliest stages of processing.

The absence of a significant group × emotion interaction ultimately precludes a strong inference of valence-specific abnormality and supports this interpretation of a generalized hypervigilance. This generalized early hypervigilance could be linked to disruptions in neurobiological systems governing motivation and attention, including aberrant salience attribution to emotional stimuli. Dysfunction in the dopaminergic reward system, a core feature of MDD [14], may lead to a failure to differentially prioritize stimuli based on their affective value, resulting in a broad, undifferentiated attentional response to any emotionally salient cue, whether positive or negative. This interpretation aligns with findings of altered reward processing and reinforcement learning in depression [35]. The unexpected bias toward happy faces in MDD could be interpreted within a framework of disrupted salience processing, whereby individuals with depression might exhibit a broad, undifferentiated attentional response to any emotionally salient cue, even if this does not translate to sustained engagement. This could either be a primary deficit in automatic attentional control circuits [36] or a downstream effect of disrupted affective valuation in early attention. Alternatively, given the high comorbid anxiety in our MDD sample, this initial bias toward happy faces might also reflect hypervigilance to perceived social threat (e.g., perceiving smiles as untrustworthy or even threatening) [37], rather than solely a reward-related salience attribution.

Our analysis of the initial gaze latency (TTFF) confirmed that both groups exhibited faster orienting to threatening (fearful) faces, but no group differences were observed. This pattern of findings, reinforced by Bayesian evidence supporting the null, strengthens the conclusion that the very earliest, automatic stage of attentional capture is characterized by a general hypervigilance to salient emotional stimuli that is not uniquely disrupted in MDD. The absence of valence-specific abnormalities in both the likelihood and the speed of initial orienting suggests that the attentional anomalies in MDD may manifest at a stage immediately following this initial capture.

Contrary to hypotheses, no significant differences emerged between MDD and HC groups in the first dwell time on emotional or neutral faces. However, a more sensitive mixed model analysis of log-transformed dwell times revealed a subtle within-stimulus effect: across both groups, the first dwell time on sad faces was significantly shorter than on fearful faces. This suggests a nuanced differentiation in the initial maintenance of attention between different threat-related stimuli, with fearful faces potentially holding attention more effectively from the very first fixation than sad faces. However, the critical absence of any interaction with the group indicates that this pattern of differentiation was not abnormal in MDD. Therefore, the primary finding is clear: individuals with MDD did not differ from controls in their sustained attentional maintenance on emotional or neutral faces. This suggests that once attention is initially allocated, individuals with MDD do not differ from controls in how long they maintain focus on emotional stimuli. These findings challenge the earlier work linking depression to prolonged attention to negative stimuli [27] but align with studies reporting inconsistent maintenance biases [24,25,26]. The divergence may stem from the task design: by rigorously controlling the initial gaze position, we effectively dissociated the automatic orienting component from subsequent maintenance processes. This design ensured that participants’ attention was systematically captured at the stimulus onset, reducing the noise from random exploration in free-viewing paradigms. Additionally, the lack of maintenance differences could refine cognitive models that emphasize sustained negativity biases [2]. The lack of maintenance differences suggests that attentional abnormalities in MDD may be most pronounced during the very early, automatic stages of processing, rather than in later, more controlled stages of engagement or disengagement.

From a theoretical perspective, our measures map onto distinct cognitive processes. The first-gaze preference is widely considered an index of automatic attentional orienting, driven by the salience of stimuli and relatively impervious to top-down control. The first dwell time, while an early measure, can reflect the initial phase of controlled attentional maintenance before strategic processes like avoidance or rumination fully take hold [13,26]. These results suggest that MDD is characterized by an anomaly at the automatic capture stage—a generalized hypervigilance to emotional salience—which subsequently normalizes. This could either be a primary deficit in automatic attentional control circuits [38] or a downstream effect of disrupted affective valuation on early attention.

Several limitations must be acknowledged. First, this study did not differentiate depression subgroups (e.g., melancholic vs. atypical depression), which may exhibit distinct patterns of attentional biases. Furthermore, our MDD sample had clinically significant levels of comorbid anxiety (mean HAMA score = 24.24). Although such comorbidity is highly prevalent in MDD, it complicates the disentangling of depression-specific versus anxiety-related effects on attentional biases. Future research should investigate whether specific clinical subtypes or comorbid symptom profiles modulate early attentional engagement with emotional stimuli. Second, the exclusive use of facial stimuli limits the generalizability of findings to other types of negative emotional cues (e.g., dynamic expressions, emotional scenes, or words). Future studies could employ ecologically valid stimuli to test the robustness of these temporal dynamics. Third, while our design isolated initial orienting, the use of a central fixation may have influenced the naturalistic attentional scan path. Furthermore, the medication status of participants in the MDD group was not controlled for, which may influence attentional processing.

Our findings offer clear implications for future research and clinical translation. Future studies should (1) utilize dynamic and complex stimuli; (2) incorporate neuroimaging to link these temporal attentional stages to neural circuits; (3) adopt longitudinal designs to track attentional changes with treatment; and (4) investigate clinical subtypes and moderators. For clinical practice, our results question the utility of therapeutic approaches that target sustained maintenance (e.g., prolonged exposure) and instead highlight the potential of stage-specific interventions. The early, automatic orienting phase may be a more fruitful target for attention bias modification (ABM). Pre-registered trials could investigate whether ABM protocols designed to specifically train early attentional disengagement from negative stimuli or engagement with positive stimuli are more effective if they are contingent on the initial orienting response.

In summary, our findings refine the understanding of attentional biases in MDD. We found evidence for a generalized hypervigilance to emotional stimuli during initial orienting but not for valence-specific abnormalities or sustained maintenance deficits. These results suggest that attentional abnormalities in MDD may be more nuanced in the automatic, rapid allocation of attention to emotional salience, potentially due to the disrupted interplay between affective and attentional control networks. These results underscore the importance of dissecting the temporal dynamics of attention to inform more targeted and effective cognitive interventions for depression.

## Figures and Tables

**Table 1:** Demographic and medication information of the MDD and HC groups.

	MDD (*n* = 61)	HC (*n* = 47)
Age (years), mean ± SD	25.97 ± 6.43	26.09 ± 4.13
Gender (female), *n* (%)	46 (75.4%)	32 (68.1%)
Hamilton Depression Rating Scale, mean ± SD	24.30 ± 4.46	0.40 ± 0.97
Hamilton Anxiety Rating Scale, mean ± SD	24.24 ± 5.49	0.85 ± 1.20
Age of onset (years), mean ± SD	24.03 ± 7.13	-
Recurrent MDD, *n* (%)	36 (59%)	-
With any psychotropic medications, *n* (%)	22 (36.1%)	-
With any antidepressants, *n* (%)	22 (36.1%)	-
With any antianxiety medications, *n* (%)	5 (8.2%)	-
With benzodiazepines, *n* (%)	8 (13.1%)	-
With mood stabilizer, *n* (%)	0 (0%)	-

**Table 2:** Eye-tracking data quality and exclusion metrics by group.

Metric	HC	MDD
Final N	47	61
Excluded Participants	4 (7.8%)	15 (19.7%)
- Excluded for comorbid diagnosis	-	10
- Excluded for HAMD score	-	2
- Excluded for poor eye-tracking	-	3
- Excluded for mental health concerns	4	-
Median Visual Angel Error	0.36 ± 0.18	0.41 ± 0.16
Mean Valid Trial Rate	94.2% ± 5.3%	94.6% ± 4.7%
Mean Blink/Sample Loss Rate	6.0% ± 7.5%	6.9% ± 6.4%

Note. Definition of “poor eye-tracking”: Participants were excluded for this reason if they had valid data in <70% of trials across all conditions or if the overall data quality was too poor to reliably parse fixations (e.g., constant loss of signal).

**Table 3:** Results of the generalized linear mixed model (logistic regression) for initial gaze preference.

Fixed Effects	Estimate	Std. Error	z	*p*	Odd Ratio	95% CI
(Intercept): HC, Fear	−0.372	0.062	−6.043	<0.001	0.689	[0.611, 0.777]
Group: MDD	−0.044	0.060	−0.735	0.462	0.957	[0.851, 1.076]
Emotion: H	0.263	0.073	3.588	<0.001	1.301	[1.127, 1.502]
Emotion: S	0.153	0.074	2.072	0.038	1.165	[1.008, 1.346]
Random Effects:		Variance	Std. Dev			
Subject (intercept)		4.00 × 10^−14^	2.00 × 10^−7^			
Stimulus (intercept)		5.80 × 10^−14^	2.41 × 10^−7^			

Note. Emotion H = Emotion Happy; Emotion S = Emotion Sad.

## Data Availability

The relevant data and analysis codes for this study have been uploaded to the Open Science Framework (OSF), and the link is https://osf.io/pqzmx/ (Accessed on 5 September 2025).

## Supplementary Materials

The following supporting information can be downloaded at https://www.mdpi.com/article/10.3390/jemr18060072/s1: Figure S1: The Q-Q plot of residuals for the logged first dwell time on emotional (left) and neutral (right) faces.; Table S1: The ANOVA result of reaction time on Group and Emotion; Figure S2: The GPower subject number estimation results.

## Abbreviations

The following abbreviations are used in this manuscript:

RT	Response Time
MDD	Major Depressive Disorder
HC	Healthy Control
HAMD	Hamilton Depression Rating Scale
HAMA	Hamilton Anxiety Rating Scale
CFAPS	Chinese Facial Affective Picture System
ANOVA	Analysis of Variance
BF	Bayes Factor
SD	Standard Deviation
CIs	Confidence Intervals
