# Supplementary material for: Initial and Sustained Attentional Bias Toward Emotional Faces in Patients with Major Depressive Disorder
Source: J Eye Mov Res. 2025 Dec 1;18(6):72. doi: 10.3390/jemr18060072 (PMC12734090; doi:10.3390/jemr18060072)
Supplement: Supplementary file 1 [file jemr-18-00072-s001.zip › jemr-3769326-supplementary.pdf]

Supplementary materials

Figure S1: The Q-Q plot of residuals for the logged first dwell time on emotional (left) and neutral (right) faces.

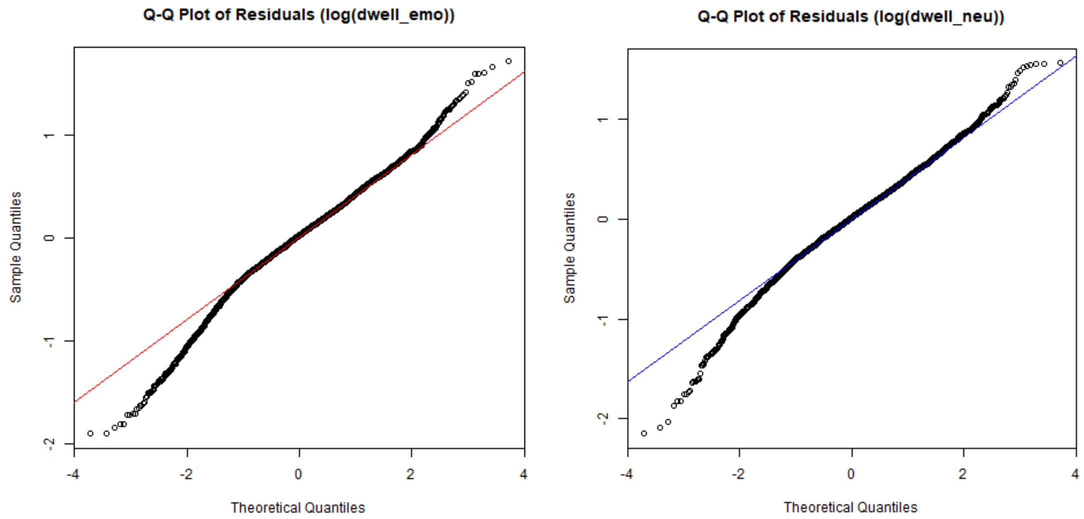

Table S1: The ANOVA result of reaction time on Group and Emotion

Effect	df	MSE	F	ges	p
Group	1, 108	257059.17	2.59	.021	.110
Emotion	1.71, 184.57	17741.58	0.30	< .001	.706
Group × Emotion	1.71, 184.57	17741.58	1.31	.001	.270

Note. df: Degree of freedom; MSE: Mean Squared Error; F: the F statistic; ges: Generalized Eta-Squared.

Figure S2: The GPower subject number estimation results

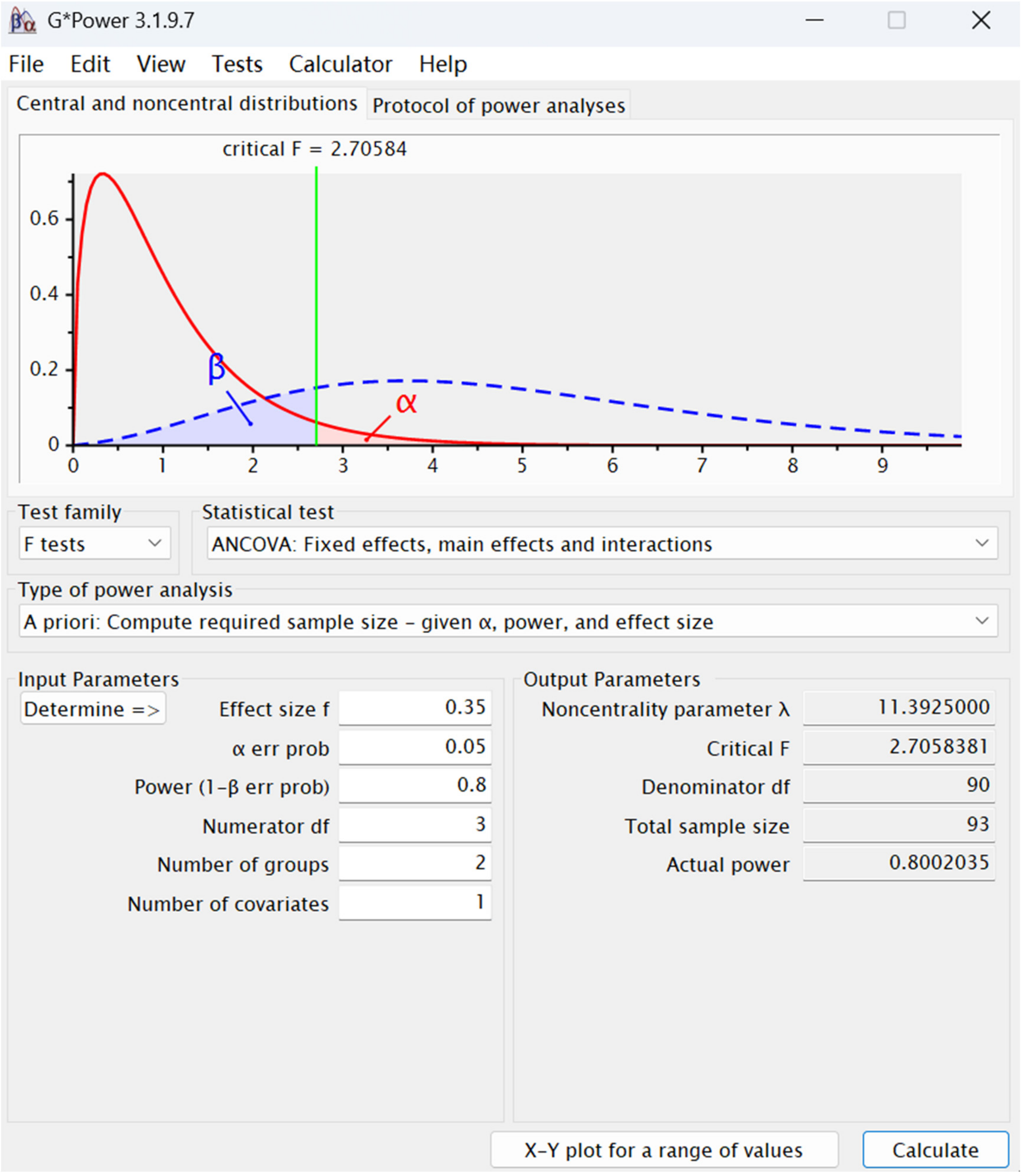
G*Power 3.1.9.7
File Edit View Tests Calculator Help
Central and noncentral distributions
Protocol of power analyses
critical F = 2.70584
β
α
Test family
F tests
Statistical test
ANCOVA: Fixed effects, main effects and interactions
Type of power analysis
A priori: Compute required sample size – given α, power, and effect size
Input Parameters
Determine =>
Effect size f 0.35
α err prob 0.05
Power (1–β err prob) 0.8
Numerator df 3
Number of groups 2
Number of covariates 1
Output Parameters
Noncentrality parameter λ 11.3925000
Critical F 2.7058381
Denominator df 90
Total sample size 93
Actual power 0.8002035
X–Y plot for a range of values
Calculate
